# First report on seroepidemiology of *Toxoplasma gondii* infection in pigs in Central Ethiopia

**DOI:** 10.1186/s12917-015-0384-y

**Published:** 2015-03-13

**Authors:** Endrias Zewdu Gebremedhin, Mulisa Megerssa Kebeta, Mebratu Asaye, Hagos Ashenafi, Vincenzo Di Marco, Maria Vitale

**Affiliations:** Department of Veterinary Laboratory Technology, Ambo University, Faculty of Agriculture and Veterinary Sciences, P. O. Box 19, Ambo, Ethiopia; Jigjiga University, College of Veterinary Medicine, P.O. Box 307, Jijiga, Ethiopia; Gondar University, Faculty of Veterinary Medicine, P.O. Box 196, Gondar, Ethiopia; Department of Parasitology and Pathology, Addis Ababa University, College of Veterinary Medicine and Agriculture, P.O. Box 34, Debre Zeit, Ethiopia; Italian National Reference Centre for Toxoplasmosis at Istituto Zooprofilattico Sperimentale della Sicilia A. Mirri, Palermo, Italy

**Keywords:** *Toxoplasma gondii*, Seroprevalence, Risk factors, Pig, Central Ethiopia

## Abstract

**Background:**

*Toxoplasma gondii* is one of the most widely prevalent cyst forming Apicomplexan parasites with significant impact on animal production particularly in sheep, goats and pigs. The objectives of this cross-sectional study were to estimate the seroprevalence and to assess risk factors of *Toxoplasma gondii* infection in pigs. A systematic random sampling technique was used to collect 402 blood samples from pigs in Central Ethiopia. Direct Agglutination Test (DAT) was used to test sera. A questionnaire survey was made to assess potential risk factors and knowledge of farm attendants about toxoplasmosis.

**Results:**

An overall seroprevalence of 32.1% [95% confidence interval (CI): 27.6%-36.9%] was found. Multivariable logistic regression analysis showed that extensively managed pigs (39.7%) are nearly twice (adjusted odds ratio [aOR]:=1.91, 95% CI: 1.01, 3.63) at higher risk of acquiring toxoplasmosis than intensively managed pigs (30.5%). Pigs supplied with feed containing animal byproducts had nearly four times (OR = 3.84, 95% CI: 2.01, 7.36) higher risk of acquiring *T. gondii* infection. Most of the farm attendants had little knowledge of health risks due to cats, neither to human nor to animals. Absence of rodent control, high neonatal mortality and history of abortion were found among herds of the studied pig farms.

**Conclusions:**

*T. gondii* infections in pigs are wide spread. Extensive management systems and pig feed types containing animal byproducts are independent predictors of *T. gondii* seropositivity. The high seroprevalence suggests that pigs might serve as an important source of *T. gondii* infection for people. This is the first report of seroepidemiology of *T. gondii* infection in pigs in Ethiopia. Further studies are warranted for designing appropriate prevention and control strategies.

## Background

Pigs are important for nutrition and economic growth in many countries of the world [1]. In Ethiopia, the pig industry is still in its infancy and the population was estimated to be 29,000 [2]. In the past years adequate emphasis was not given for pig production in Ethiopia. Unlike other livestock distribution, swine farms are found predominantly in the central part of the country, particularly, in Addis Ababa and its surroundings.

*Toxoplasma gondii* is one of the most widely prevalent cyst forming Apicomplexan parasites. Cats and other felids play a crucial role in the epidemiology of toxoplasmosis as the definitive hosts, through shedding of millions of oocysts when infected [1,3]. Human beings and other warm-blooded animals, which serve as intermediate hosts, are infected primarily by ingesting food or water contaminated with sporulated oocysts or by ingesting meat that contain tissue cysts of *T. gondii* [3,4]. The parasite has a significant impact on animal production particularly in sheep, goats and pigs. *Toxoplasma gondii* causes mortality in pigs, especially neonatal pigs [1]. Most pigs acquire *T. gondii* infection postnatally by ingestion of oocysts from a contaminated environment or ingestion of infected tissues of animals [1,3]. Some pigs become infected prenatally by transplacental transmission of the parasite [1]. One-third of the human population is infected by this parasite worldwide. The routes of transmission differ in human populations depending on social culture, eating habits, religion, and/or environmental factors [5].

In Ethiopia Christian Orthodox and Muslim religion followers, which account for 43.5% and 33.9% of the total population, respectively, do not consume pork [6]. Although studies on *Toxoplasma gondii* seroprevalence and associated risk factors have been documented in different species of livestock in Ethiopia, [7-17] there are no studies conducted on seroepidemiology of *T. gondii* infection in pigs and the role of pigs in the transmission of toxoplasmosis to humans remains unknown in Ethiopia. Therefore, the objectives of this study were to estimate the seroprevalence and to assess the potential risk factors of acquiring *T. gondii* infection in pigs in selected areas of Central Ethiopia.

## Methods

### Description of the study areas

**Figure 1:**
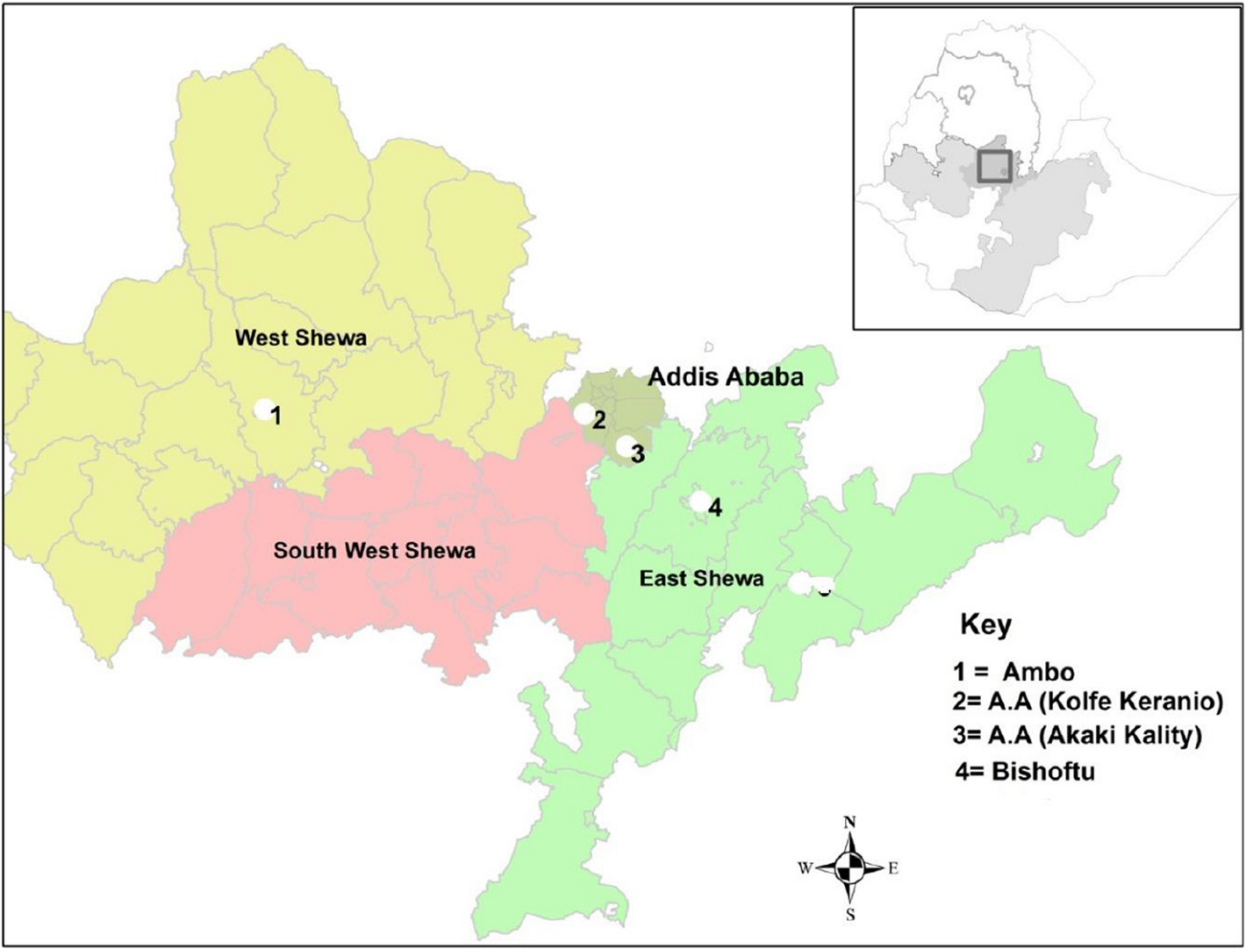
**Study areas.**

The study was carried out in four purposively selected areas located in Central Ethiopia, namely Akaki Kaliti sub-city, Kolfe Keraniho sub-city (Addis Ababa), Bishoftu and Ambo (Figure 1). The selection of the study areas was on the basis of availability of pig farms and accessibility.

Addis Ababa is the capital city of Ethiopia located at longitude 38° 44′- 24″ E and latitude 9°1′ 48″ N. The altitude is from 2,200 to 3300 meters above sea level (masl). The annual rainfall is 800–1100 mm and the mean annual maximum and minimum temperature is about 21°C and 27°C, respectively [18].

Bishoftu is found in Adea district of East Shewa Zone of Oromia Regional State at a distance of 45 kms from Addis Ababa. Bishoftu town is situated at a longitude of 38°59′ E to 38.983°E and latitude of 08° 45′N to 8.750°N at an elevation of 1920 masl. The average rainfall is about 839 mm, while the mean minimum and maximum temperatures recorded for 27 years ranged from 7.9°C to 28°C with an overall average of 18.5°C [19].

Ambo district is found in West Shewa Zone of Oromia Regional State. The town is located at 8° 59′ 0″ N, 37° 51′ 0″ E at an elevation of 2101 masl. The mean annual temperature and rainfall of the town over 30 years (1981–2010) is about 18.64°C and 968.7 mm, respectively [20].

### Study populations

The study was conducted in pigs originating from the four purposively selected areas in Central Ethiopia. The majorities of the pig populations studied were from the Bishoftu area, where many large and small scale commercial pig farms are concentrated. In the Bishoftu area, pigs are mainly kept under an intensive production system. In the Addis Ababa area, both intensive and extensive pig management systems are common, while in the Ambo area pigs are kept under an extensive management system. All pigs of more than three months of age belonging to both sexes (male and female) were considered for the purpose of this study. Age was determined using tooth eruption patterns [21].

### Study designs and sample size

A cross-sectional study design was carried out from August, 2013 to May, 2014 to collect blood samples from pigs using a systematic random sampling technique. In the absence of previous studies on *T. gondii* infection in pigs in Ethiopia the required sample size was calculated by considering 50% expected prevalence (P) and 95% confidence interval (Z = 1.96) with a 5% desired absolute precision (d) using the formula N = (Z)^2^*P*(1-*P*)/*d*^*2*^ [22]. The calculated sample size (N) was 384; however, 402 pigs were sampled for the study. The total sample size was allocated to study site as follows. Bishoftu [246 animals: 139 young, 107 adult from six intensive farms], Ambo [57 animals: 13 young, 44 adult from two extensive farms], Akaki Kality [24 animals: 10 young and 14 adult from 4 extensive farms] and Kolfe Keraniho [75 animals: 8 young, 67 adult from one intensive farm].

### Sample collection and transportation

Blood samples were aseptically collected from ear vein using sterile vacuum tubes without anti-coagulant, labeled and were immediately transported in an ice box with ice packs to the Ethio-Belgium Laboratory of the College of Veterinary Medicine and Agriculture of Addis Ababa University, Bishoftu for serological testing. Blood samples were centrifuged at 3200 RPM for 10 minutes; the separated sera were transferred to eppendorff tubes and kept at −20°C until serologically assayed.

### Laboratory investigation

#### Direct Agglutination Test (DAT)

*Toxoplasma gondii*-specific IgG antibodies were detected by a direct agglutination test (DAT) (Toxo screen DA, biomerieux®, France) following the procedure described by the manufacturer of the kit. Sera were assayed at a screening dilution of 1/40 and 1/4000 in order to avoid the false negative results that might occur at low dilutions when using sera with high antibody titers. A titer of 1: 40 or 1: 4000 or both was considered indicative of *T. gondii* exposure. Sedimentation of antigen at the bottom of the well and clear agglutination above half of the well at either dilutions were recorded as negative and positive results respectively. Positive and negative controls were included in each test.

### Questionnaire survey

A structured questionnaire was administered for each farm owner regarding herd size, age, sex (female/male), management system (intensive/extensive), feed type (with/without animal products), presence of domestic cats (yes/no), housing of domestic cats (totally outdoor/mixed [indoor and outdoor]), presence of rodents on the farm (yes/no), access for pigs to dead animals (yes/no), water source (pipe/mixed [pipe, river and pond]), presence of dogs (yes/no) and history of reproductive failure (abortion, still birth, neonatal mortality, mummification) were recorded during sample collection to assess potential risk factors for *T. gondii* infection in pigs. Furthermore, two people were interviewed from each of the 13 farms to assess farm attendants’ or owners’ knowledge about zoonotic importance of toxoplasmosis.

### Data analysis

Data generated from the questionnaire survey and laboratory investigations were recorded and coded using Microsoft Excel spreadsheet (Microsoft Corporation) and analyzed using the STATA version 11.0 for Windows (Stata Corp. College Station, TX, USA). The seroprevalence was calculated as the number of seropositive pigs divided by the total number of pigs samples tested. Confidence limits for the proportions were established by exact binomial test with 95% confidence intervals (CI). All the variables assessed were categorical and variables with more than two categories were transformed into indicator (dummy) variables. To identify predictors of seropositivity, first the association of the potential risk factors (origin, age, sex, management system, presence of domestic cat, presence of dog, way of life of domestic cat, pig feed type, access to dead animal and water source) were analyzed by univariable logistic regression. Then, all non-collinear variables with *P*-value ≤ 0.25 in univariable logistic regression analysis were included in the final multivariable logistic regression model to construct the likely model (*P* < 0 .05). The model was reduced by backwards elimination of non-significant variables (*P* > 0.05) based on a likelihood ratio test to define the model that would best fit the data. In line with this, (with the exception of the management system, type of pig feed, presence of dog and age) all variables were dropped due to collinearity. Variables with small and incomparable frequencies in the contingency table were not considered. A significance level of α = 0.05 was used.

### Ethics considerations

The animal part of the research project was reviewed and approved by the ethical committee for animal experimentation of the College of Veterinary Medicine and Agriculture, Addis Ababa University, Ethiopia. All animals were handled strictly in accordance with good animal practice. Verbal consent of owner’s permission to obtain samples from pigs and to participate in the questionnaire and interviews part of the study was approved by College of Health Sciences, Addis Ababa University (No. DRERC 001/11/MLS).

## Results

### Questionnaire

Among 26 farm attendants interviewed during the study 14 (53.8%) have completed university education, whereas only 2 (7.7%) were illiterate. Sixteen (61.5%) and 18 (69.2%) of the study participants do not have adequate knowledge about the role of cats in transmitting zoonotic diseases to humans and animals respectively (Table 1).

**Table 1:** **Educational status and knowledge about health risks of cats among farm attendants (n = 26)**

**Variable**	**Category**	**Frequency (%)**
Level of education	Illiterate	2 (7.7)
	Primary School	4 (15.4)
	Secondary school	6 (23.1)
	University	14 (53.8)
Knowledge of health risk to humans due to cats	Yes	10 (38.5)
	No	16 (61.5)
Knowledge of health risk to animals due to cats	Yes	8 (30.8)
	No	18 (69.2)

Out of 13 pig farms considered for this study, 12 (92.3%), 10 (76.9%) and 9 (69.2%) farms reported the presence of neonatal mortality, weak birth and history of abortion in their farms, respectively. Nine farms (69.2%) did not practice rodent control in their farms (Table 2).

**Table 2:** **Farm characteristics and reproductive problems in pig farms in study area (n = 13)**

	**Frequency (%)**	
Variable	Yes	No
History of abortion (s)	9 (69.2)	4 (30.8)
Presence of stillbirth	8 (61.5)	5 (38.5)
Presence of neonatal mortality	12 (92.3)	1 (7.7)
Presence fetal mummification	9 (69.2)	4 (30.8)
Presence of weak births	10 (76.9)	3 (23.1)
Contamination of stored animal feeds by cat faeces	12 (92.3)	1 (7.7)
Presence of feral cats	13 (100)	0 (0)
Presence of separate house for cats	0 (0)	13 (100)
Pig mortality in farms	10 (76.9)	3 (23.1)
Presence of rodent control	4 (30.8)	9 (69.2)

### Seroprevalence

The seroprevalence of IgG antibodies to *T. gondii* in pigs was 32.1% (129/402) (95% CI: 27.6%-36.9%). The highest animal level seroprevalence was observed in Kolfe Keraniho Sub-city (57.3%) followed by Akaki Kaliti Sub-city (45.8%), Ambo (33.3%) and Bishoftu (22.8%). These results are significantly different between study areas (*P* < 0.001). The seroprevalence of *T. gondii* infection was higher in extensively managed pigs (39.7%) than those pigs under intensive management (30.5%) (Table 3).

**Table 3:** **Results of univariable analysis of potential risk factors associated with** ***Toxoplasma gondii*** **seropositivity**

**Variable category**		**Number tested**	**Test Pos. (%)**	χ^**2**^	***P*** **value**	**Univariable OR (95% CI)**	***P*** **value**
Origin				33.9	<0.001		
	Bishoftu	246	56 (22.8)			1.0	
	Ambo	57	19 (33.3)			1.7 (0.91-3.17)	0.098
	Akaki Kaliti	24	11 (45.8)			2.87 (1.22-6.76)	0.016
	Kolfe Keraniho	75	43 (57.3)			4.56 (2.64-7.87)	<0.001
Sex				0.43	0.511		
	Female	240	74 (30.8)			1.0	
	Male	162	55 (34.0)			1.15 (0.75-1.76)	0.511
Age				2.27	0.039		
	<12 months	170	45 (26.5)			1.0	
	≥12 months	232	84 (36.2)			1.58 (1.02-2.43)	0.039
Management				2.18	0.140		
	Intensive	334	102 (30.5			1.0	
	Extensive	68	27 (39.7)			1.5 (0.87-2.57)	0.142
Presence of cats				0.03	0.864		
	No	61	19 (31.2)			1.0	
	Yes	341	110 (32.3)			1.05 (0.58-1.89)	0.864
Presence of dogs				16.2	<0.001		
	No	157	32 (20.4)			1.0	
	Yes	245	97 (39.6)			2.56 (1.61-4.08)	<0.001
Housing of domestic cats				0.26	0.607		
	Totally outdoor	138	42 (30.4)			1.0	
	Mixed*	264	87 (33)			1.12 (0.72-1.75)	0.607
Pig feed type				26.96	<0.001		
	Without animal product	327	86 (26.3)			1.0	
	With animal product	75	43 (57.3)			3.77 (0.24-6.33)	<0.001
Access to cadaver				10.22	0.001		
	No	230	59 (25.7)			1.0	
	Yes	172	70 (40.7)			1.99 (1.30-3.04)	0.001
Water source				0.28	0.599		
	Pipe	333	105 (31.5)			1.0	
	Mixed**	69	24 (34.8)			1.16 (0.67-2.0)	0.599
Rodent presence				1.35	0.245		
	No	12	2 (16.7)			1.0	
	Yes	390	127 (32.6)			2.41 (0.52-1.18)	0.260

* = indoor and outdoor, ** = pipe, river and pond.

### Risk factors

For the univariable analysis the variable “presence of contamination of pig feed with cat faeces” was not considered in logistic regression because of smaller frequency. History of abortion, presence of still birth, neonatal mortality, fetal mummification, weak births and pig mortality were all excluded from the analysis because of lack of information on individual animals. The potential risk factors assessed for seropositivity of pigs are shown in Table 3.

Pigs reared on farms having access to dogs (OR = 2.56, 95% CI: 1.61, 4.08) had significantly higher seroprevalence than pigs reared on farms where dogs were not present. Similarly, origin, age, type of pig feed and access of pigs to cadavers were significantly associated with *T. gondii* seropositivity. The presence of rodents on farms, gender, source of water and management systems for pigs did not significantly influence the seropositivity of animals (*P* > 0.05) (Table 3).

The data obtained from the questionnaire surveys showed that all pigs in the study area had access to feral cats. Moreover, separate cat housing systems were not practiced for domestic cats. Domestic, as well as feral cats, have free access to pig farms. These variables were not subjected to statistical analysis as they were common to all farms (Table 3).

For multivariate logistic regression non-collinear variables with *P*-value <0.25 in univariable logistic regression were considered. Selection of one of the collinear variables was done on the basis of biological grounds to reason out scientifically the hypothesized risk factors in a better way. Sex, presence of domestic cats, housing of domestic cats, source of water and presence of rodents were variables excluded from the final model due to univariable *P*- value > 0.25. Origin and access for pigs to cadavers were removed due to collinearity. Finally, the management system, age category, type of pig feed and presence of dogs were entered into multivariable logistic regression models and the results are depicted in Table 4.

**Table 4:** **Results of multivariable logistic regression analysis of predictors of** ***T. gondii*** **infection in pig of study area**

**Variable category**	**Adjusted OR (95% CI)**	***P*** **-value**
Management	1.91 (1.01-3.63)	0.047
Type of pig feed	3.84 (2.01-7.36)	<0.001
Presence of dog	1.48 (0.84-2.61)	0.172
Age	1.12 (0.68-1.84)	0.665

In the multivariate logistic regression analysis, management system and type of feed were the only risk factors significantly associated (*P* < 0.05) with pig seroprevalence (Table 4). The risk of acquiring toxoplasmosis in pigs raised on extensively managed farms was 1.91 times higher than pigs raised under intensive management. Type of feed was also a risk factor associated with toxoplasmosis, in that pigs fed on additional animal byproducts (slaughterhouse leftovers, dairy products (whey)) as feed supplement had a higher chance of acquiring *T. gondii* infection than pigs that were not supplemented with such animal products.

## Discussion

### Questionnaire

The survey results indicate that most of the respondents have little knowledge and awareness regarding the role of cats in the transmission of diseases either to humans (61.5%) or animals (69.2%), respectively. Therefore, this may contribute towards the transmission of various zoonotic diseases including toxoplasmosis. Several studies have indicated that contact with cats’ faeces is a major risk factor for pregnant women in acquiring *T. gondii* infection [23,24]. Furthermore, animals and humans can be infected by ingesting soil, water, or plant materials contaminated with *T. gondii* oocysts shed by infected cats [25]. In order to understand the transmission dynamics of zoonotic parasitic infections to humans, it is essential to have knowledge on the life cycle and prevalence of infections in both domestic and wild animals [26]. It is believed that people with a background of formal education are much more dedicated to health care than those without formal education and in most of the cases; they can have a tendency to gather information about health risk factors and its prevention [24].

The results of the questionnaire survey revealed widespread presence of neonatal mortality, history of abortion and presence of weak births among pig herds of the study areas. Previous studies showed that *T. gondii* infections result in tremendous problems for livestock husbandry and cause huge economic losses due to reproductive wastage [27,28]. Among domestic animals, abortion due to *T. gondii* in goats and sheep and high mortality rates in swine populations are widely noted [1,29]. Mortality due to *T. gondii* infection is more common in young pigs than in adult pigs [30]. Pigs infected transplacentally with *T. gondii* may be borne premature, dead, or weak, or may die soon after birth [31]. Recent reports from Korea have also demonstrated higher abortion rates (up to 44%) and unusually high sow mortality rates (up to 19%), that were primarily associated with toxoplasmosis [32]. The causes of reproductive failure in pigs have never been investigated in Ethiopia, however non-infectious (heat stress, hormonal disturbance, toxic chemicals, mineral and vitamin deficiencies) and infectious (porcine reproductive and respiratory syndrome virus, porcine parvovirus, pseudorabies virus, Japanese B encephalitis virus, classical swine fever virus, *Leptospira* spp, and *Brucella suis*) causes have been reported elsewhere [33].

Even though, the role of rodents as a reservoir of *T. gondii* has not yet been studied in domestic animals, including pigs in Ethiopia, the absence of rodent control on pig farms in this study might be an important factor for the transmission of pig toxoplasmosis. Kijlstra *et al.* [34] suggested that rodents can act as a reservoir for transmission of *T. gondii* and inadequate rodent control is considered to play a key role in *T. gondii* infection of pigs.

### Seroprevalence

This is the first comprehensive survey of *T. gondii* infection in pigs in Ethiopia. The overall prevalence of the infection was found to be 32.1%. The result of the present study was much higher than the reports from Brazil (12.5%) using indirect fluorescent antibody tests (IFAT) [35], Central Italy (16.14%) using IFAT [36], Southern Italy (16.3%) [37] using Enzyme-Linked Immunosorbent Assay (ELISA), Serbia (9.2%) [38] and Portugal (15.6%) [39] using modified agglutination test (MAT). In contrast, the present result was lower than the report from Egypt (56.6%) using MAT [40]. However, this result is comparable with the reports from Northwestern Taiwan (28.8%) [41], South-west China (30.6%) [42], Peru (32.3%) [43], the Netherlands (31%) [44] and Switzerland (32%) [45].

The difference in seroprevalence between the present study and the aforementioned reports might be associated with the type of serological technique employed [46], cut-off values used, sample sizes and sampling techniques [47], type of rearing (free range vs. confinement) [1,3] climatic variations, density of feline and rodent control on farms [48,49]. The high seroprevalence of *T. gondii* in the present study might be associated with farm management systems and access of free roaming cats on pig farms. According to Arko-Mensah *et al.* [50] the risk factors influencing the prevalence of antibodies against *T. gondii* in Ghana was the age of the animals, breed, environmental conditions, and management practices.

The highest seroprevalence of *T. gondii* infection was found in pigs from the Kolfe Keraniho sub-city of Addis Ababa (57.3%) while the lowest seroprevalence was from the Bishoftu area (22.8%). The likely reasons for differences in seroprevalence among study areas might be associated with the difference in environmental temperature and management of pigs. Kolfe Keraniho is characterized by a warm and moist agro-climate compared to the mid highlands of Ambo and the relatively warmer and drier climatic condition in the midland of the Bishoftu area. The influence of the environment on the epidemiology of toxoplasmosis has been well documented [4]. It has been suggested that a warm and moist climate is more frequently associated with a high prevalence of *T. gondii* infection than hot dry ones [3]. This is due to the fact that the oocysts of *T. gondii* survive and sporulate easier in a moist and humid environment [37] whereas a dry climate has a deleterious effect on the persistence and dissemination of *T. gondii* oocysts, which in turn is likely to decrease the chance of oocyst survival, generally resulting in a low prevalence of toxoplasmosis [3].

### Risk factors

Even though the seroprevalence of toxoplasmosis was not significantly associated with the presence of cats in the pig units/ farms, all pig farms in the study areas were accessed for either domestic or feral cats. Domestic cats have no separate housing system and have frequent outdoor access. Feed storage areas are easily accessed by feral and domestic cats leading to the possible contamination of pig feed and the environment with *T. gondii* oocysts shed in cats’ faeces. This finding corroborates the report of Fajardo *et al.* [51] suggesting that *T. gondii* infected cats spending time looking for rats in feed storage locations can defecate on the feed leading to its contamination with oocysts. Presence of cats is considered as one of the main risk factors for seropositivity in pigs, especially in animals kept in outdoor facilities. This is due to the fact that only a few oocysts are sufficient to produce infection in pigs [1,36]. Furthermore, most farm workers do not use boots and coveralls or footbaths before entering stables, thus contributing to the introduction of the oocysts collected from the environment into the pig units. This could mask the influence of exposure to cats as a risk factor [36]. Therefore, the transmission of toxoplasmosis to pigs might rely on the presence of cats, either feral or domestic, on the pig farm. In America, the presence of cats on farms was confirmed to be an important risk factor for seropositivity of *T. gondii* infection in sows [52].

There is meager data regarding the epidemiology of *T. gondii* infection in cats in the study area. A recent report showed a very high seroprevalence (85.4%) of *T. gondii* and oocyst shedding (22.2%) in feral cats in Addis Ababa, Ethiopia, suggesting a high environmental contamination with oocysts which might also serve as a source of infection for other animals [8,12]. Studies have shown a high seroprevalence of *T. gondii* infection in pigs on farms with high soil contamination by *T. gondii* oocysts and presence of a high number of cats [53,54]. Therefore, the possible risk factors for pig infection with *T. gondii* in the present study area might be associated with exposure of pig farms to cats.

The final multivariable logistic model showed that the types of pig feed containing animal byproducts and extensive management systems are independent predictors of toxoplasmosis. Studies have indicated that the prevalence of *T. gondii* infection in pigs influenced by management systems may be as high as 68% in poorly managed non-confinement systems (free range pigs), where pigs are kept in properties with no or limited technological developments for effective sanitary measures. Prevalence may also vary strongly from one locality to another due to differences in certain ecological factors [1,55]. Earlier research done in the Netherlands reported a significantly higher risk of seropositivity for *Toxoplasma* antibodies in free range pigs than for those in an intensive pig unit [56,57]. In contrast, a study in Switzerland showed that conventional fattening of pigs under confined conditions and free-range pigs surprisingly exhibited the same prevalence (2.0%; 95% CI: 0.2–7.0%) [58]. A possible explanation for the high seropositivity of extensively managed pigs in the present study might be related to scavenging in areas contaminated with either cat fecal materials containing oocysts, or feeding on cadavers containing infective bradyzoites. Moreover, free-roaming behavior in extensively managed pigs favor contact with rodents and the probability of ingestion of infective cysts contained in their tissues [59,60].

In this study, the type of feed consumed by pigs was more likely to be a notable source of *T. gondii* infection as it frequently includes slaughter byproducts, which might contain tissue cysts. In addition, feed supplements, such as concentrates, forages, household leftovers, raw vegetables, and fruits might be contaminated with cat faeces containing oocysts.

Although presence of dogs on pig farms had no significant association with toxoplasmosis in the final model, it was significantly associated with *T. gondii* infection during univariable analysis. Many dogs were seen roaming around pig feeds (household leftovers, animal byproducts like cow whey, slaughterhouse leftovers). These dogs might contaminate pig feeds with oocysts of *T. gondii* from cat faeces. Although dogs do not produce *T. gondii* oocysts like cats, it has been suggested that outdoor dogs might contribute to *Toxoplasma* transmission in two ways: fur contamination and oocysts re-shedding near houses following ingestion of infected cat feaces [61,62]. Other studies also revealed that under laboratory conditions, dogs have been shown to eliminate viable oocysts after ingesting sporulated oocysts in cat feaces. Moreover, the fur of dogs that have come in contact with cat faeces may be a vector for transmission of oocysts to humans [63]. Data in US swine operations indicated that it is difficult to eliminate *T. gondii* infection from swine herds which allow access of cats or dogs to swine facilities [64].

Univariable logistic regression analysis of the present study revealed significantly higher seroprevalence (36.2%), (OR = 1.58; 95% CI: 1.02-2.43) in pigs of ≥ 12 months of age compared to those pigs <12 months of age. Similar findings were reported from elsewhere [37,47]. A recent study from Slovakia also identified that the seroprevalence of toxoplasmosis in sows (4.26%) was twice as high in slaughter pigs (2.06%) [65]. The relatively higher seroprevalence in pigs of ≥ 12 months of age as compared to pigs of < 12 months of age might be due to the longer contact time of older animals with a potentially infected environment containing *T. gondii* oocysts and/or tissue cysts [37].

Although pork is staple food widely eaten in many countries of the world, traditional Ethiopian food does not use any pork as most Ethiopians have historically adhered to Islam, the Ethiopian Orthodox Church, or Judaism, all of which prohibit eating pork. However, with increased population growth and urbanization, consumption of pork in becoming popular in urban areas among foreigners and a few Ethiopians (protestant). Generally, there is no raw pork consumption habit in Ethiopia but there are possibilities of unsafe handling and preparation of pork (e.g. humbuggers) leading to the consumption of undercooked smoked pork hence there is chance of acquiring *T. gondii* infection.

## Conclusions

In conclusion, the study showed that *T. gondii* infection in pigs is widespread. Extensive management systems and pig feed types containing animal byproducts are independent predictors of *T. gondii* seropositivity. The high seroprevalence suggests contamination of the environment with *T. gondii* oocysts from freely moving cats on the premises of pigs and the likely role of pigs as a potential source of *T. gondii* infection for humans. Feeding of pigs with heat treated animal products and further studies to unravel the role of toxoplasmosis of pigs in causing reproductive failures and its zoonotic transmission are imperative.

## Abbreviations

Masl: Meters above sea level
°C: Degree celsius
RPM: Revolutions per minute
DAT: Direct agglutination test
CI: Confidence interval
IFAT: Indirect fluorescent antibody test
ELISA: Enzyme linked immunosorbent assay
MAT: Modified agglutination test
OR: Odds ratio
aOR: Adjusted odds ratio
